# A280 HEPATOBILIARY DISEASE IN INDIVIDUALS LIVING WITH FRAGILE X SYNDROME: A CASE SERIES

**DOI:** 10.1093/jcag/gwad061.280

**Published:** 2024-02-14

**Authors:** A Alalool, G Hirschfield, K Leung

**Affiliations:** Department of Medicine, University of Toronto, Toronto, ON, Canada; Medicine, Division of Gastroenterology & Hepatology, University Health Network, Toronto, ON, Canada; Medicine, Division of Gastroenterology & Hepatology, University Health Network, Toronto, ON, Canada

## Abstract

**Background:**

Fragile X syndrome is a genetic disorder known to cause intellectual disability through decreased or absent FMR protein (FMRP). Basic science studies have identified potential associations with hepatobiliary inflammation. Clinical reports of liver disease in Fragile X syndrome are however infrequent.

**Aims:**

We describe 3 individuals living with Fragile X syndrome and chronic liver disease.

**Methods:**

A case series and literature review were performed.

**Results:**

The 1^st^ person is a woman with concurrent Fragile X and autosomal recessive polycystic kidney disease, initially seen by Hepatology at 18 y.o. with evidence of hepatic fibrosis on abdominal ultrasound with normal liver enzymes (Table 1). She subsequently developed liver cirrhosis and portal hypertension, as well as biliary duct dilatation seen on MRI. Her overall presentation was thought to be in keeping with Caroli syndrome and congenital hepatic fibrosis, and she is currently pending genetic screening for hepatorenal ciliopathy.

The 2^nd^ person is a woman with Fragile X syndrome and ulcerative colitis, who initially presented to Hepatology at 24 y.o. with mixed liver enzyme abnormalities. Lab tests over time revealed immunology suggestive of autoimmune hepatitis (AIH) (Table 1). Subsequent investigations demonstrated primary sclerosing cholangitis (PSC) with cirrhosis. Her disease is currently managed as AIH/PSC overlap syndrome with ursodeoxycholic acid (UDCA), tacrolimus, mycophenolate, and budesonide.

The 3^rd^ person is a man with Fragile X syndrome who initially presented to Hepatology at 17 y.o. with acute hepatitis with AIH immunology (Table 1). Liver biopsy at the time demonstrated findings suggestive of early PSC as well as possible AIH. He continued to have progressive cirrhosis as well as overt PSC on subsequent abdominal imaging and is currently managed as AIH/PSC overlap syndrome with UDCA, azathioprine, and budesonide.

A review of literature revealed that asides from known intellectual, behavioral, and physical effects of Fragile X syndrome, there are minimal reports of other associated medical complications including liver problems or autoimmune conditions including IBD. There are however reports of autoimmunity in those with altered FMR1 (Fragile X Mental Retardation 1) gene mutations.

**Conclusions:**

These 3 cases of liver disease in Fragile X highlight young age of presentation for chronic liver disease with both biliary and immune-mediated characteristics, along with progression to cirrhosis. Additional review for liver manifestations in people living with Fragile X would help understand if this association is incidental or causal.

Investigations of individuals with Fragile X syndrome and liver disease

**Funding Agencies:**

None

**Table:** 

Patient ID	Initial Blood Chemistries	Immunology	Liver Pathology Characteristics	MRI Features	Fibroscan
1	Plts normal AST 28 ALT 30 ALP 89 GGT 49 Alb 41 INR normal (2010)	AMA neg. ANA 1:80 pos. (speckled & cytoplasmic) SMA 1:160 pos. Immunoglobulins normal (2022)	Ductal plate malformation, in keeping with congenital hepatic fibrosis. Biliary-type fibrosis variable, severe in areas, suggesting a superimposed biliary process, possibly Caroli’s syndrome. (2022)	Cirrhotic liver with features of portal hypertension, multiple regions of mild-moderate intrahepatic biliary dilatation with predominant peripheral distribution and evidence of strictures. (2022)	21.3 kPa (2023)
2	AST 390 ALT 793 ALP 652 (2009)	AMA neg. ANA ampersand:003E1:640 (homogenous) anti-SSAampersand:003E8 ASMA 1:80 anti-SLA high pos. Anti-Ro medium pos. IgG 27.5 (2018)	Chronic hepatitis with moderate portal inflammation (lymphoid infiltrate with occasional plasma cells & neutrophils), moderate interface inflammation, moderate lobular necro-inflammation with zone 4 concentric fibrosis (mild). Focal duct injury by lymphocytes. (2017)	No dominant stricture or convincing cholangiocarcinoma. Increased scattered areas of probable perfusion abnormalities throughout the hepatic parenchyma. Extra and intrahepatic biliary strictures and dilatations related to PSC. Stable/mildly decreased burden of intraductal stones / sludge. (2023)	75 kPa (2022)
3	AST 1197 ALT 1111 ALP 408 Bilirubin 31 INR 1.5 (2015)	ASMA pos. 1:160 ANA 1:160 IgG 33.6 (2015) ANA 1:640 homogenous+speckled ASMA, LKM – neg. (2021)	Portal lymphoplasmacytic infiltrate and interface hepatitis With lobular activity, fibrosis (2/4), focal acute cholangitis, findings are compatible with AIH, acute cholangitis raises possibility of early PSC. (2015)	Appearance of mild intrahepatic bile duct dilatation, intraductal calculi and peribiliary inflammation. No evidence of cholangiocarcinoma or dominant stricture. Cirrhotic liver with stable findings of portal hypertension including splenomegaly and varices. No definite evidence of hepatocellular carcinoma. (2023)	48 kPa (2022)

